# The Yin and the Yang of Prediction: An fMRI Study of Semantic Predictive Processing

**DOI:** 10.1371/journal.pone.0148637

**Published:** 2016-03-24

**Authors:** Kirsten Weber, Ellen F. Lau, Benjamin Stillerman, Gina R. Kuperberg

**Affiliations:** 1 Department of Psychiatry and the Athinoula A. Martinos Center for Biomedical Imaging, Massachusetts General Hospital, Harvard Medical School, Charlestown, Massachusetts, United States of America; 2 Department of Psychology and Center for Cognitive Science, Tufts University, Medford, Massachusetts, United States of America; 3 Max Planck Institute for Psycholinguistics, Nijmegen, The Netherlands; 4 University of Maryland, Department of Linguistics, College Park, Maryland, United States of America; University Zurich, SWITZERLAND

## Abstract

Probabilistic prediction plays a crucial role in language comprehension. When predictions are fulfilled, the resulting facilitation allows for fast, efficient processing of ambiguous, rapidly-unfolding input; when predictions are not fulfilled, the resulting error signal allows us to adapt to broader statistical changes in this input. We used functional Magnetic Resonance Imaging to examine the neuroanatomical networks engaged in semantic predictive processing and adaptation. We used a relatedness proportion semantic priming paradigm, in which we manipulated the probability of predictions while holding local semantic context constant. Under conditions of higher (versus lower) predictive validity, we replicate previous observations of reduced activity to semantically predictable words in the left anterior superior/middle temporal cortex, reflecting facilitated processing of targets that are *consistent* with prior semantic predictions. In addition, under conditions of higher (versus lower) predictive validity we observed significant differences in the effects of semantic relatedness within the left inferior frontal gyrus and the posterior portion of the left superior/middle temporal gyrus. We suggest that together these two regions mediated the suppression of unfulfilled semantic predictions and lexico-semantic processing of unrelated targets that were *inconsistent* with these predictions. Moreover, under conditions of higher (versus lower) predictive validity, a functional connectivity analysis showed that the left inferior frontal and left posterior superior/middle temporal gyrus were more tightly interconnected with one another, as well as with the left anterior cingulate cortex. The left anterior cingulate cortex was, in turn, more tightly connected to superior lateral frontal cortices and subcortical regions—a network that mediates rapid learning and adaptation and that may have played a role in switching to a more predictive mode of processing in response to the statistical structure of the wider environmental context. Together, these findings highlight close links between the networks mediating semantic prediction, executive function and learning, giving new insights into how our brains are able to flexibly adapt to our environment.

## Introduction

Graded probabilistic prediction is thought to play a crucial role in language processing. We use multiple types of contextual information to predict upcoming information at multiple grains and levels of representation. Inputs that confirm these predictions are processed more efficiently than inputs that are not predicted (see [1] for a recent review), and inputs that disconfirm these predictions allow us to adapt to our ever-changing communicative environments [2–5]. In this study, we used a relatedness proportion semantic priming paradigm, together with functional Magnetic Resonance Imaging (fMRI), to explore the neuroanatomical networks engaged in semantic predictive processing and adaptation.

There is evidence from the event-related potential (ERP) literature that the neural signatures associated with confirmed and disconfirmed semantic predictions may be distinct [5–7]. Specifically, while the N400—a negative-going ERP waveform that peaks between 300-500mm from the onset of a given target word—is thought to reflect semantic facilitation when the target *confirms* prior semantic predictions (e.g. [8,9]), a later set of ERP components, which tend to peak after the N400 time window, appear to be selectively modulated when the target *disconfirms* medium or high probability semantic predictions ([5,7,10,11]; see also Discussion section).

ERPs, however, do not have the spatial resolution to determine which neuroanatomical regions are engaged in semantic predictive processing. We know from many fMRI studies that single word semantic contexts can modulate activity within a network of regions that includes the anterior temporal cortex, the left posterior superior/middle temporal gyrus/sulcus (post-S/MTG), and the left inferior frontal gyrus/sulcus (left IFG) (e.g. [12–14]). Many of these same regions are also modulated by the semantic predictability of sentence contexts (e.g. [15–20]; see [21] for a review), and they are each thought to play distinct functional roles in semantic processing. Specifically, the anterior temporal cortex may act as a hub that maps highly distributed conceptual-semantic features onto amodal semantic representations [22–24]; the left post-S/MTG may play a more specific role in lexico-semantic processing, that is, mapping phonological or orthographic word-form on to semantic features [21,25–27], while the left IFG has been implicated in the suppression of semantic distractors, as evidenced by both lesion [28–32] and fMRI [13,33–39] studies (see [40] for a review). Whether these regions play a role in *predictive* semantic processing, however, remains unclear.

One reason why it has thus far been difficult to address this question is methodological: the sluggish hemodynamic response evoked by a given target word cannot be easily deconvolved from that evoked by its context (the only way to do this would be to use very long intervals between prime and target, or to jitter the interval between prime and target—both introducing many psychological confounds). And because predictable and non-predictable sentence and discourse contexts often differ along multiple dimensions, including the lexical properties of their component individual words, the semantic relatedness between these words and the syntactic structure of the context, any effects of semantic predictability on the hemodynamic response might have nothing to do with semantic predictive processing per se, but might simply reflect the effects of these other factors (see [41] for a more detailed discussion).

To address this issue, we used a paradigm that has been used for many years to study semantic predictive processing using single word contexts—the s*emantic priming relatedness proportion paradigm* ([42–46], see [47] for a review). Rather than manipulating the probability of encountering a target word by varying the semantic predictability of a preceding local sentence or discourse context, this paradigm achieves the same goal by varying the proportion of predictable associated versus non-predictable unrelated trials within the broader contextual environment. There is now a large body of evidence from both behavioral [42–46] and ERP [41,48,49] studies showing that participants modulate the strength of their semantic predictions, based on the predictive validity of the broader environmental context. Thus, in a block that contains a relatively higher proportion of associated (versus unrelated) word pairs, participants will use the prime to predict upcoming semantic features with greater strength than in a block that contains a relatively lower proportion of associated (versus unrelated) word pairs. The reason for this is that, so long as probabilistic predictions are based on the statistical probabilistic knowledge of the broader contextual environment, and so long as they have some expected utility, prediction should maximize the chances of optimal task performance (see [4,50] for discussion).

Obviously, the semantic priming relatedness proportion paradigm is less naturalistic than sentence or discourse processing paradigms that vary the predictability of the local context. However, it has the advantage of allowing the local semantic context to be held constant while varying the probability of a particular semantic prediction being confirmed or disconfirmed by an incoming target word. This means that neural processing of exactly the same set of target words, preceded by exactly the same set of contexts (prime words), can be contrasted across blocks of high versus low predictive validity. In this way, Relatedness (semantically associated versus unrelated word pairs) can be fully crossed with Predictive Validity (higher proportion of semantically associated word pairs versus lower proportion of semantically associated word pairs) in a 2 x 2 design (see Methods for further details). Thus, the neuroanatomical regions engaged in semantic predictive processing can be isolated while avoiding the types of confounds described above.

In previous work, we have used this paradigm in conjunction with ERP and MEG techniques. In an initial ERP study [41], we established that, just as in sentence processing paradigms, the N400 to target words was selectively attenuated when semantic predictions, based on the prime, were fulfilled: semantically associated target words in high predictive validity blocks showed more semantic facilitation than the same set of associated targets in low predictive validity blocks ([41]; see also [48,49]). In a second ERP/MEG study (supplemented by a preliminary fMRI analysis), we used source localization methods to show that the differential activity to semantically associated versus unrelated targets within the N400 time window under conditions of higher but not lower predictive validity, localized to the left anterior temporal cortex [51]. This finding was consistent with a previous PET study that used a similar relatedness proportion priming paradigm and reported an effect of relatedness proportion in this anterior temporal region [52], although, because PET does not allow for an event-related design, the authors were unable to probe differential neural activity associated with the priming itself (the modulation of activity to associated versus unrelated word pairs).

Our previous ERP/MEG work using this paradigm suggested that the anterior temporal cortex was modulated by *confirmed* semantic predictions [51]. However, it left open the question of whether the other regions described above—the left IFG and post-S/MTG—also contribute to semantic predictive processing. In our ERP study [41], we showed some evidence of an ERP effect that was more prolonged and that had a more anterior distribution than the N400 effect, which was selectively evoked by unrelated target words in the high versus low predictive validity blocks. This effect may have reflected the suppression of medium probability semantic predictions that were disconfirmed by the unrelated targets (e.g. [9,53]). As noted above, semantic suppression has also been linked to activity within the left IFG. Although our MEG study showed no hint of modulation within the left IFG within the N400 time window, we were unable to examine neural activity past the N400 time window because there was too much ocular artifact in the later part of the evoked response.

In the present study, we used the same relatedness proportion semantic paradigm in conjunction with fMRI—a technique that has better spatial resolution than either ERP or MEG. Our first aim was to determine which neuroanatomical regions were specifically modulated by semantic predictive processing. On the basis of our previous MEG/ERP findings, we expected to see increased modulation within the left anterior superior/middle temporal cortex (left ant-S/MTG) in the higher versus the lower predictive validity blocks, reflecting enhanced predictive semantic facilitation. We hypothesized that, under conditions of higher predictive validity, we would also see more activity to unrelated versus associated targets within the left IFG, recruited to suppress unfulfilled semantic predictions, and within the left post-S/MTG, which is often co-activated with the left IFG (e.g. [13,39,54], and which may reflect increased lexico-semantic activity to unpredicted target words.

The second goal of this study was to begin to explore the neuroanatomical relationships between semantic prediction and adaptation. As discussed above, manipulating the proportion of associated word pairs within a block leads participants to modulate the strength (or certainty) of their semantic predictions. In fact, the relationship between adaptation and prediction is reciprocal: there is a large body of evidence from models of animal learning [55,56], connectionist models [57,58], and probabilistic Bayesian models [59,60] suggesting that prediction itself may be a computational mechanism that drives adaptation. Within at least some of these frameworks, the magnitude of the prediction error modulates the *rate* of adaptation (e.g. [61,62]).

In the brain, the main region implicated in mediating between prediction error and adaptation is the anterior cingulate cortex (ACC). While the precise role of the ACC is unclear, one proposal is that it continually monitors changes in statistical contingencies between stimuli [63] and stimulus-response mappings [64], using this information to weight the degree to which current prediction error influences adaptation to these statistical contingencies [65,66]. Learning itself may be mediated by a lateral superior/middle prefrontal-subcortical network to which the anterior cingulate cortex is closely connected, including the middle and superior lateral prefrontal cortices [63,67], and the thalamus and basal ganglia [68–73].

To explore the relationships between the regions mediating prediction and adaptation in the present study, we used a functional connectivity analysis [74,75]. If the left IFG and post-S/MTG are indeed recruited in response to unfulfilled predictions in the high predictive validity block, and if the anterior cingulate cortex mediates between prediction error and adaptation, then we should see more functional connectivity across these three regions under conditions of higher versus lower predictive validity.

## Methods

### Participants

Participants were 26 students recruited from universities in the Boston area, with an average age of 23.54 (age range: 20 to 30; 11 female). Three additional subjects were run in the study but were subsequently excluded from analysis: two because of artifacts in the data and one because of technical issues during scanning. All participants were native speakers of American English (who did not grow up speaking another language), without any prior history of neuropsychiatric disorders, and all were right-handed, as assessed using the Edinburgh Handedness Inventory [76]]. This study was carried out with the explicit review and approval of the Partners Human Research Committee and Massachusetts General Hospital IRB (study protocol #2010P001683). Participants gave written informed consent and were compensated for taking part in the study in accordance with the approved IRB protocol.

### Stimuli and Overall Experimental Design

The experimental design and stimuli have been previously described in detail [41,51]]. Briefly, the fMRI design comprised two experimental factors, Relatedness (semantically associated versus semantically unrelated) and Predictive Validity (higher predictive validity versus lower predictive validity), which were fully crossed in a 2 x 2 design. The Relatedness manipulation was achieved by comparing associated word pairs (all with a forward association strength of .5 or higher on the University of South Florida Association Norms [77] and unrelated word pairs (created by randomly redistributing the primes across the target items, and checking by hand to confirm that this did not accidentally result in any related pairs). The Predictive Validity manipulation was achieved by adding different numbers of associated or unrelated filler word pairs to two blocks, such that the overall proportion of associated and unrelated word pairs differed across these two blocks. In the higher predictive validity block, 50% of word pairs (200/400) were associated, while in the lower predictive validity block, only 10% of the word pairs (40/400) were associated. Importantly, each participant saw a core set of 40 controlled and counterbalanced items in each of the four conditions (associated and unrelated) in each of the two predictive validity blocks, and no participant saw the same word twice. The subsequent analysis was done on this core set of 40 items per condition. Forward association strength between prime and target and log frequency for both prime and target, did not significantly differ between test items in each block. Eighty of the unrelated filler word pairs included an animal word, either in the prime or target position. These were necessary for participants to carry out a semantic monitoring task, as discussed below.

In the higher predictive validity block, where 50% of the word pairs were associated, the cumulative probability of encountering a given set of semantic features (e.g. a set of semantic features corresponding to the word, chair) following a prime word like table can be roughly estimated as the association strength of table (0.75 according to the Florida Association Norms [77]) multiplied by the broader probability of encountering an associated word in that block (0.5), i.e. 0.375. In the lower predictive validity block, however, where only 10% of the word pairs were associated, the cumulative probability of encountering the same set of semantic features following the same prime is only 0.075. Thus, participants should use the prime to predict upcoming semantic features with greater certainty in the higher than in the lower relatedness proportion block.

### Stimuli Presentation and Task

Stimuli were projected onto a screen in white 20-point uppercase Arial font. Each trial began with a fixation cross, presented at the center of the screen for 200ms, followed by a 200ms blank screen. The prime word was then presented for 500ms, followed by a 100ms blank screen, and then the target word was presented for 900ms, followed by a 100ms blank screen. Thus, the stimulus onset asynchrony between prime and target was 600ms to encourage controlled rather than automatic semantic priming [47]. Participants were instructed to press a button on a handheld response box with their left thumb as quickly as possible when they saw a name of an animal. As noted above, the animal words appeared on 80/400 of the filler trials (there were no animal words in the experimental trials). This task ensured that participants processed the words semantically while at the same time not drawing their explicit attention to semantic relationships between primes and targets.

Following previous studies, the lower predictive validity block was always presented first, followed by the higher predictive validity block. This ensured that, during the low proportion block, participants had not already adapted to using the prime as a strong predictor of the target. Although this potentially introduced a potential confound of block order, most of the low-level variables that would normally be associated with trial order, such as lower attention and lower motivation, would predict reduced rather than increased effect sizes in the higher versus lower predictive validity block, going against our hypotheses.

Each block constituted 400 trials, which was divided into four runs, each of 100 trials. The order of stimuli within each run was randomized using the OptSeq algorithm to improve deconvolution of the hemodynamic response [78]. For this purpose fixation trials of different lengths (varying from between 2 and 10 seconds) were added.

The fMRI experiment was carried out as part of a larger study in which participants were also recruited to participate in a separate MEG session in which we used the same relatedness proportion design reported in [51]. The order of the two sessions was counterbalanced, and two completely distinct stimuli sets were created such that no primes or targets were repeated across sessions for any participants.

### Structural and functional MRI data acquisition

Structural and functional magnetic resonance images were acquired using a 3T Siemens Trio scanner using a 32-channel head coil. FMRI data were acquired over eight runs (4 runs per predictive validity block), each lasting for approximately 5 minutes. In each run, 130 functional volumes (36 axial slices (AC-PC aligned), 3mm slice thickness, .3mm skip, 200mm field of view, in-plane resolution of 3.125mm) were acquired with a gradient-echo sequence (repetition time = 2s, echo time = 25ms, flip angle = 90deg, interleaved acquisition). In addition, at the beginning and end of the scanning session, we acquired two T1-weighted high-resolution structural images (1mm isotropic multi-echo MPRAGE: TR = 2.53s, flip angle = 7, four echoes with TE = 1.64ms, 3.5ms, 5.36ms, 7.22ms). We used the higher quality structural scan (based on visual inspection) for the subsequent analysis.

### Data analysis

Pre-processing as well as the first and second level analyses of the fMRI data were conducted in Statistical Parametric Mapping 8 (SPM8, www.fil.ion.ucl.ac.uk/spm), supplemented by additional add-on toolboxes [79,80]].

### Preprocessing

The first four images in each run were discarded to ensure that transient non-saturation effects did not affect the analysis. The next step was to detect spikes and interpolate these bad slices from surrounding images (using the ArtRepair toolbox). On average 0.3% of slices (range 0 to 1.4%) were removed and interpolated. Then, images were slice-time corrected and the volumes were realigned to the first images of each run and then to each other. The functional images were co-registered to the structural image by co-registering the mean functional image to the structural MPRAGE. The anatomical images were segmented into grey and white matter, and the spatial normalization parameters acquired during this step were used to normalize the functional images. Finally, the images were smoothed with a 10mm FWHM Gaussian kernel.

### Standard functional activation analyses

#### First level statistical analysis: Individual participants

We modeled the data using a design matrix in which each of the two blocks—lower predictive validity and higher predictive validity—had four runs (following the experimental design described above). Each run had the following regressors: two for each level of Relatedness (associated and unrelated), one for the unrelated filler trials (the four higher predictive validity runs had an additional regressor for the associated fillers), and two for the probe animal-word filler trials (one for probe pairs in which the prime was an animal word, and one for trials in which the target was an animal word).

The trials were modeled from the start of the prime word until the end of the target word, i.e. the total time for each trial (1.8 s) was taken as its duration. All regressors were convolved with a canonical hemodynamic response function. Temporal derivatives [81]] were included for all conditions. The realignment parameters for movement correction were also included in the model. In addition, we used additional regressors to covary for excessive movement at time points where the image intensity was greater than 3*SD or composite motion >0.5 mm. These covariates were created using the ART toolbox [80]]. On average the additional regressors were added for less than 5% of the time points.

We defined four contrasts to take to the second level for random effects group analysis:

1. [Higher Predictive Validity including Semantically Associated and Unrelated regressors (contrast value 1) versus implicit baseline (contrast value 0)]
2. [Lower Predictive Validity including Semantically Associated and Unrelated regressors (contrast value 1) versus implicit baseline (contrast value 0)].
3. [Higher Predictive Validity and Semantically Associated regressors (contrast value 1) versus Higher Predictive Validity and Semantically Unrelated regressors (contrast value -1)]
4. [Lower Predictive Validity and Semantically Associated regressors (contrast value 1) versus Lower Predictive Validity and Semantically Unrelated regressors (contrast value -1)]

#### Second level statistical group analysis

We created two repeated measures ANOVA models for the effects of Relatedness and the effect of Predictive Validity. The first model was created to look at the effect of all word pairs relative to the implicit baseline as well as the main effect of Predictive Validity and consisted of the within-subject effect (26 subject regressors) as well as one regressor for the effect of Higher predictive validity (versus the implicit baseline; contrast (a)) and another for the effect of lower predictive validity (versus the implicit baseline; contrast (b)).

The second model was created to investigate the main effect of Relatedness and interaction between Relatedness and Predictive Validity. This consisted of the within-subject effect (26 subject regressors) and one regressor each for the effect of Relatedness in the Higher predictive validity (contrast (c)) and the lower predictive validity blocks (contrast (d)). Within these models statistical parametric maps (SPMs) were created for the t-statistics of the effects of interest, namely the main effects of Predictive Validity, Relatedness and the interaction between the two as well as word pairs compared to implicit baseline.

To home in on effects specifically related to semantic and lexico-semantic processing, we defined three *a priori* regions of interest to be used for small volume correction [82]] (a) the *left anterior superior/middle temporal gyrus* (left ant-S/MTG), which, as noted in the introduction, may act as a hub, mapping distributed conceptual-semantic features on to amodal semantic representations, and that has been shown in recent semantic priming MEG studies to be sensitive to highly automatic [24]] and predictive [51]] semantic facilitation in the N400 time window; (b) the *left inferior frontal gyrus* (left IFG), which has been associated with top-down semantic suppression [33,34,40]]; and (c) the *left posterior superior/middle temporal gyrus* (left post-S/MTG), which has been associated with lexico-semantic processing [21]]. All three regions were defined functionally as spheres of 5mm radius around a peak MNI coordinate, revealed using the search term, ‘semantic’ on Neurosynth software for automatic metanalysis ([83]], values retrieved 2015/01/07): the left ant-S/MTG peak coordinate: -58,-6,-10; the left IFG peak coordinate (in pars triangularis): -50,21,14, and the left post-S/MTG peak coordinate: -54,-40,4.

We report whole-brain effects at a voxel-level threshold of p<0.005, and either (a) a cluster-level familywise error-corrected (FWE) threshold of p<0.05 or (b) a small volume correction FWE-corrected at the peak of *a priori* regions of interest described above (the three regions of interest were combined into one image for small volume correction to account for multiple comparisons across these three regions). All reported coordinates are in MNI space.

### Functional task-related connectivity analysis

In addition to activation analyses, we also carried out a hypothesis-driven connectivity analysis using the generalized context-dependent psychophysiological interactions (gPPI) toolbox [84]] to determine whether there was a difference between the higher and lower predictive validity blocks in the patterns of connectivity from two seed regions: the left inferior frontal cortex and the left anterior cingulate cortex.

#### First level statistical analysis: Individual participants

Our seed regions were the left inferior frontal cortex (IFG) and the left anterior cingulate cortex (ACC). For each of these regions, we specified a seed cluster based on relevant contrasts in the activation analyses (see Results). We entered the time series from each seed into the model as explanatory variables in each of our four conditions. Although we were only interested in the contrast between higher predictive validity versus lower predictive validity, we modelled all four conditions in order to mirror the structure of the activation analysis model described above. This gave rise to eight regressors: four interaction regressors describing connectivity from the left IFG seed in each of our four conditions (psychophysiological interactions), and four regressors corresponding to the connectivity from the left ACC seed in each of our four conditions (psychophysiological interactions). The design matrix also included regressors corresponding to activity in each of our four experimental conditions, regressors for activity in the left ACC and left IFG seeds and their interaction, and regressors for the three-way psychophysiological interactions between the left IFG, left ACC and activity in each condition across the rest of the brain.

We then defined four contrasts to take to the second level for a random effects group analysis, each modeling a psychophysiological interaction against the implicit baseline and each collapsing across the two levels of Relatedness:

1. [left IFG connectivity at Higher Predictive Validity regressors (contrast value 1) versus implicit baseline (contrast value 0)],
2. [left IFG connectivity at Lower Predictive Validity regressors (contrast value 1) versus implicit baseline (contrast value 0)],
3. [left ACC connectivity at Higher Predictive Validity regressors (contrast value 1) versus implicit baseline (contrast value 0)],
4. [left ACC connectivity at Lower Predictive Validity regressors (contrast value 1) versus implicit baseline (contrast value 0)],

#### Second level statistical analysis: Functional connectivity group analysis

To determine whether connectivity from each of these regions (the left IFG and left ACC) differed between the higher and lower predictive validity blocks (a main effect of Predictive Validity), we used two design matrices—one for each seed. Each model consisted of a within-subject effect (thus 26 subject regressors) and two regressors that collapsed across Relatedness: one for connectivity (psychophysiological interaction) at higher predictive validity (contrasts (a) or (c)) and one for connectivity at lower predictive validity (contrasts (b) or (d)). Within these models statistical parametric maps (SPMs) were created for the t-statistics of the effects of interest, the main effects of Predictive Validity.

We report effects at a voxel-level threshold of p<0.005 and either (a) a cluster-level FWE-corrected threshold of p<0.05, or (b) a small volume correction FWE-corrected threshold that allowed us to home in on connectivity from our seed regions to our two temporal regions of interest: the left ant-S/MTG and the left post-S/MTG. All reported coordinates are in MNI space.

## Results

### Behavioral data

The participants detected, on average, 83% of animal words in the higher predictive validity block and 84% of animal words in the lower predictive validity block (overall range: 69% to 96%). This small difference was not statistically significant, p>0.05. These data show that participants were on task and attending to the semantic features of each word.

### Standard functional activation analyses

**Fig 1:**
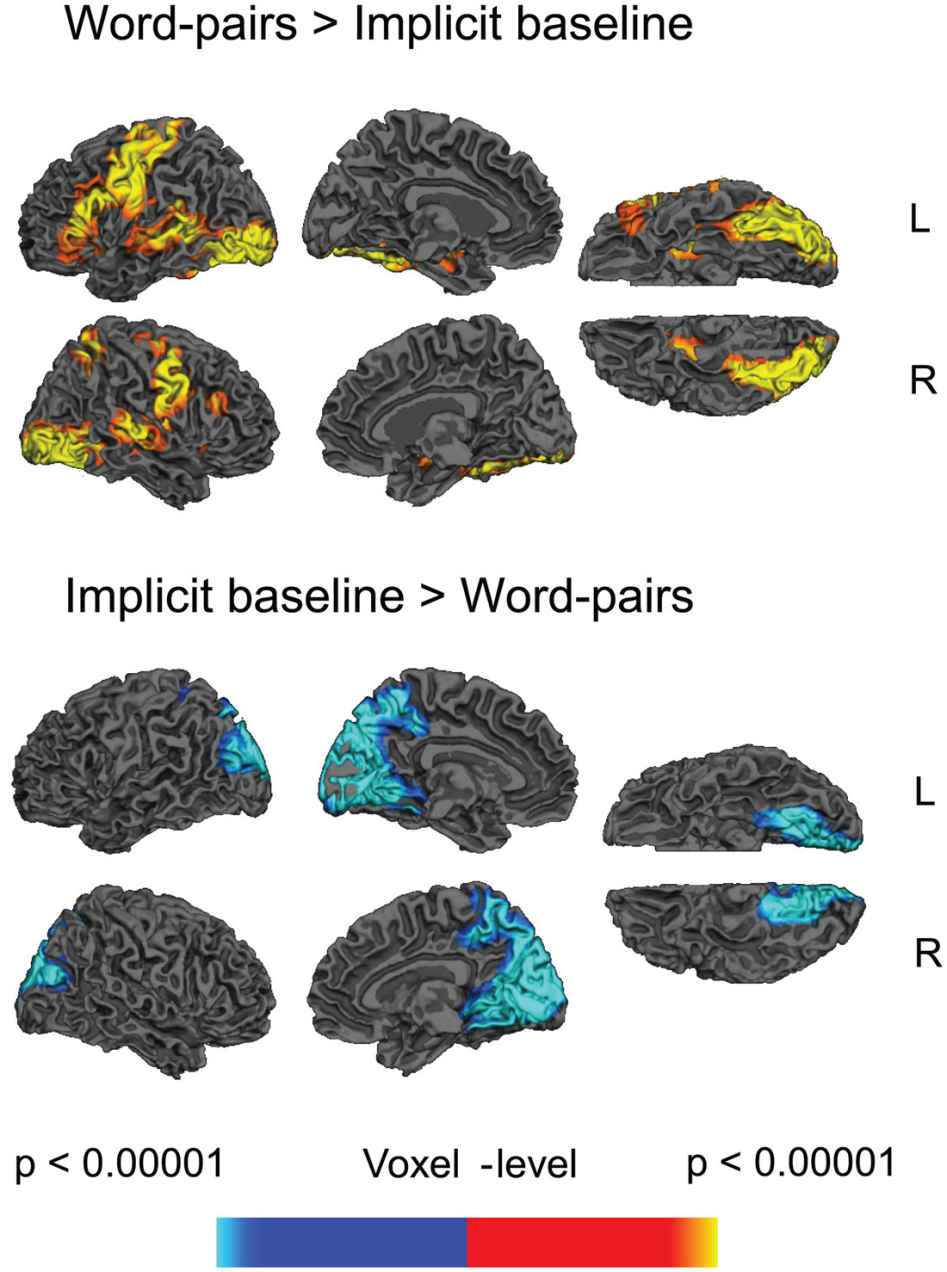
Statistical maps showing effects of word pairs versus implicit baseline. Yellow—red: more activity to word pairs than implicit baseline. Blue: less activity to word pairs than implicit baseline. Effects are shown at a voxel-level significance threshold of p<0.005, and include clusters consisting of 10 or more contiguous voxels. See Table 1 for the full list of peaks. Gray masks cover subcortical regions in which activity is displaced in the surface visualisations.

The directed contrast comparing all word pairs and the implicit baseline revealed increased activity to the word pairs across a bilateral but left lateralized network distributed across the frontal cortices (inferior frontal cortices, pre-central cortices), occipital cortices, temporal cortices (the temporal fusiform cortices and, on the left, the middle temporal cortex) and subcortical regions (left and right caudate extending through the putamen and pallidum into the thalamus). The reverse contrast showed more activity to the implicit baseline than word pairs within the occipital lobe, extending into the precuneus, see Fig 1 and Table 1. There were no clusters that showed main effects of Predictive Validity.

**Table 1:** Hemodynamic modulation: All word pairs vs. implicit baseline.

Region	R/L	BA	Peak voxel p-value	z-score	MNI (x, y, z)	Cluster extent k	Cluster level p-value (FWE corrected)
**1. Word Pairs > Implicit Baseline**							
Inferior frontal gyrus (pars triangularis)	L	44	< 0.0001	6.44	-36, 18, 22	23,912	<0.0001
Inferior frontal gyrus (pars orbitalis)	L	38/47	0.0002	3.57	-42, 30, -18		
Precentral gyrus	L	6	< 0.0001	7.44	-46, -4, 60		
Postcentral gyrus	L	3/4	< 0.0001	6.28	-58, -18, 56		
Inferior parietal lobule (supramarginal gyrus)	L	41	< 0.0001	5.53	-42, -40, 24		
Middle temporal cortex (posterior)	L	21	< 0.0001	5.74	-46, -44, 6		
Fusiform cortex (temporal)	L	37	< 0.0001	7.66	-38, -50, -16		
Fusiform cortex (occipital)	L	37	< 0.0001	∞	-38, -64, -10		
Hippocampus (anterior)	L	20	< 0.0001	4.31	-28, -12, -16		
Occipital cortex (lateral)	L	18	< 0.0001	∞	-30, -90, -8		
Basal ganglia (putamen)	L	-	< 0.0001	6.66	-26, 2, 0		
Basal ganglia (caudate)	L	-	0.0021	2.86	-8, 26, 10		
Inferior frontal gyrus (pars triangularis)	R	45	< 0.0001	4.09	40, 26, 22	10,947	< 0.0001
Precentral gyrus	R	6	< 0.0001	7.32	54, 2, 52		
Insula	R	48	0.0002	3.50	28, 30, 10		
Postcentral gyrus	R	43	< 0.0001	5.55	66, -6, 32		
Superior temporal cortex (anterior)	R	21	< 0.0001	4.92	64, -24, 2		
Hippocampus (anterior)	R	20	< 0.0001	3.93	30, -8, -16		
Basal ganglia (putamen)	R	-	< 0.0001	6.63	26, 6, 0		
Basal ganglia (caudate)	R	-	0.0008	3.16	16, 32, 12		
Thalamus	R	-	0.0012	3.04	8, 0, 0		
Fusiform cortex (temporal)	R	37	< 0.0001	5.63	36, -40, -22	5,660	0.0002
Occipital cortex (lateral)	R	19	< 0.0001	∞	34, -88, -6		
**2. Implicit Baseline > Word Pairs**							
Fusiform cortex (temporal)/Cerebellum	L	30	< 0.0001	5.29	-18, -42, -14	25,318	< 0.0001
Fusiform cortex (temporal)/Cerebellum	R	37	< 0.0001	4.93	22, -46, -12		
Precuneus	L	5/7	< 0.0001	5.46	-2, -56, 58		
Precuneus	R	5/23	< 0.0001	4.38	10, -44, 46		
Occipital cortex (calcarine)	L	17/18	< 0.0001	7.09	-6, -84, 14		
Occipital cortex (calcarine)	R	17/18	< 0.0001	7.11	4, -82, 18		
Occipital cortex (lingual)	L	18	< 0.0001	6.86	-10, -72, 2		
Occipital cortex (cuneus)	L	7/19	< 0.0001	6.14	-12, -78, 44		
Occipital cortex (cuneus)	R	19	< 0.0001	6.60	20, -88, 20		

All regions shown reached a cluster-level significance threshold (after family-wise error correction) of p<0.05. Anatomical locations, MNI coordinates, and approximate Brodmann areas (BA) correspond to the p-values and z-scores of representative peaks within each cluster. Both the AAL atlas and the SPM anatomy toolbox [85]] were used to define the anatomical regions reported. Only one peak per anatomical region is reported for each hemisphere. The cluster-level p-values indicate the cluster-level significance after family-wise error correction, and k indicates the number of contiguous voxels within each cluster.

**Fig 2:**
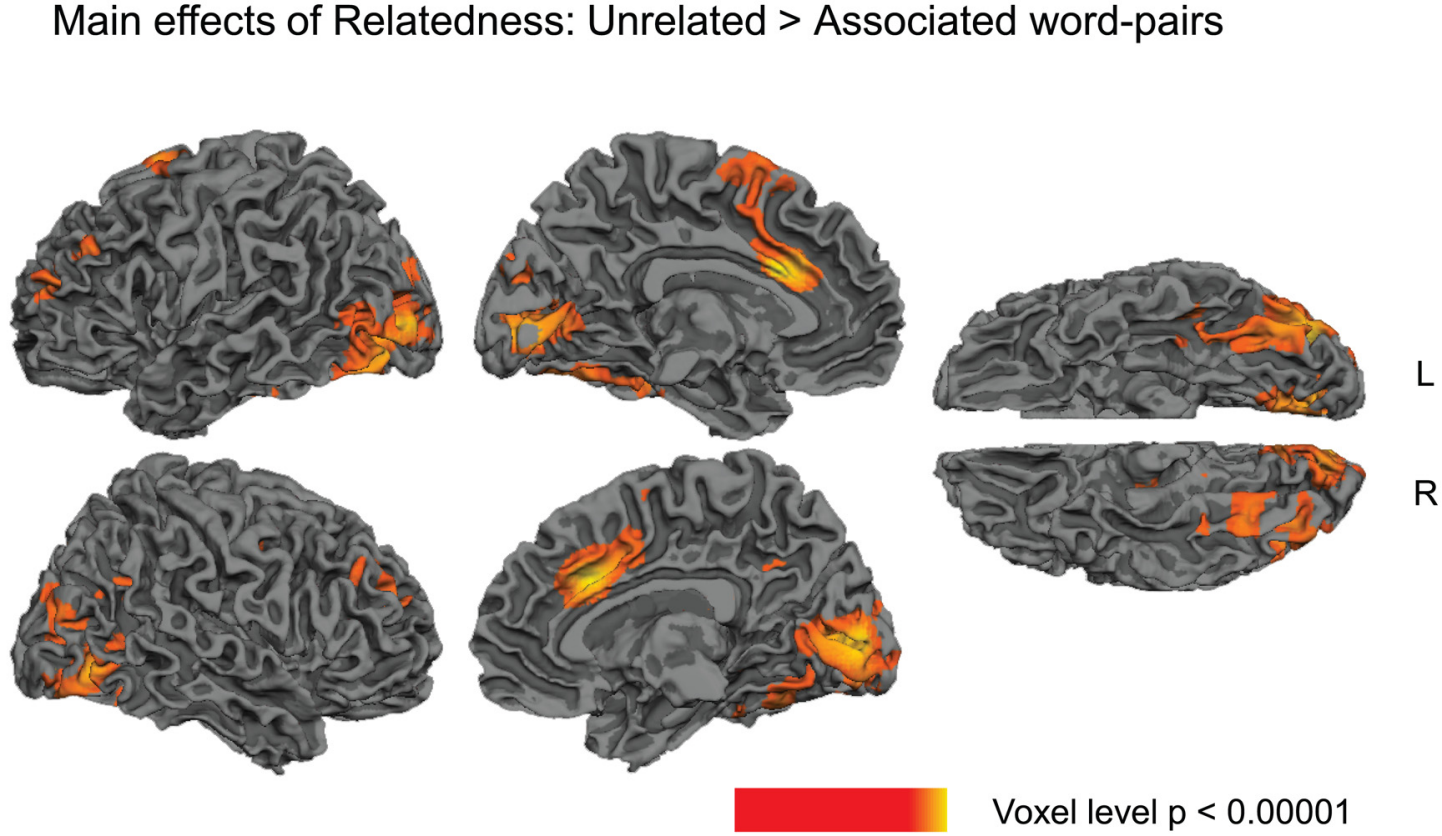
Statistical maps showing main effects of Relatedness (collapsing across higher and lower predictive validity blocks). Yellow—red: more activity to unrelated than associated word pairs. Effects are shown at a voxel-level significance threshold of p<0.005, and include clusters consisting of 10 or more contiguous voxels. See Table 2 for the list of peaks within these clusters. Gray masks cover subcortical regions in which activity is displaced in the surface visualisations.

Several clusters showed main effects of Relatedness (collapsed across higher and lower predictive validity blocks): the directed contrast between unrelated versus associated showed that there was significantly less activity to associated than unrelated word pairs (hemodynamic response suppression) within the left and right temporal fusiform gyri, extending into occipital areas, and in the left anterior/middle cingulate, extending into supplementary motor area (SMA), see Fig 2 and Table 2. No clusters, however, showed more activity to associated than unrelated word pairs (hemodynamic response enhancement).

**Table 2:** Regions showing more hemodynamic activity to unrelated than associated word pairs (collapsed across higher and lower predictive validity blocks).

Region	R/L	BA	Peak voxel p-value	z-score	MNI (x, y, z)	Cluster extent k	Cluster level p-value (FWE corrected)
**1. Unrelated > Associated word pairs**							
Middle frontal cortex	L	46	0.0006	3.23	-30, 34, 30	10,796	0.001
Supplementary motor area	L	6	0.0005	3.3	-10, 10, 70		
Anterior cingulate cortex	L	24	< 0.0001	4.05	-6, 18, 26		
Anterior cingulate cortex	R	24	< 0.0001	4.03	8, 22, 30		
Middle cingulate cortex	R	24	< 0.0001	4.06	16, 4, 38		
Middle cingulate cortex	L	24/32	0.0016	2.94	0, 4, 46		
Inferior parietal lobule (angular gyrus)	R	39	0.0005	3.31	38, -50, 30	3,838	<0.0001
Middle temporal cortex (posterior)/ Middle occipital cortex	L	19/37	0.0012	3.03	-58, -74, 10		
Precuneus	R	7/23	0.0001	3.69	20, -54, 32		
Fusiform cortex (occipital)	L	19	0.0006	3.24	-34, -64, -16		
Occipital cortex (cuneus)	L	18	0.0009	3.11	-6, -92, 26		
Occipital cortex (calcarine)	R	17	< 0.0001	3.84	6, -90, 6		
Occipital cortex (calcarine)	L	17	< 0.0001	3.72	-2, -88, 0		
Occipital cortex (lingual)	L	17	0.0006	3.23	-4, -74, 6		
Occipital cortex (lateral)	L	18	< 0.0001	3.85	-34, -90, 2		
Occipital cortex (lateral)	R	19	0.0002	3.56	40, -72, -4		
Cerebellum	L	-	0.0019	2.89	-8, -48, -34		
Cerebellum	R	-	< 0.0001	3.86	38, -38, -36		

All regions shown reached a cluster-level significance threshold (after family-wise error correction) of p<0.05. Anatomical locations, MNI coordinates, and approximate Brodmann areas (BA) correspond to the p-values and z-scores of representative peaks within each cluster. Both the AAL atlas and the SPM anatomy toolbox [85]] were used to define the anatomical regions reported. Only one peak per anatomical region is reported for each hemisphere. The cluster-level p-values indicate the cluster-level significance after family-wise error correction, and k indicates the number of contiguous voxels within each cluster.

In the whole brain analysis (voxel-level, p<0.005), there was an interaction between Relatedness and Predictive Validity (for the directed contrast between: [(Higher Predictive Validity and Semantically Unrelated—Higher Predictive Validity and Semantically Associated)—: (Lower Predictive Validity and Semantically Unrelated—Lower Predictive Validity and Semantically Associated)]) in two of the three regions hypothesized, and both these effects reached significance on small-volume correction: the left IFG (dorsal portion: Z = 2.71, p_FWE_ < .05) and the left post-S/MTG (Z = 2.93, p_FWE_ = .01). The IFG effect appeared to be bilateral (right Z = 3.42) although, as we had no *a priori* reason to look at the right IFG, this did not survive small volume FWE correction.

**Fig 3:**
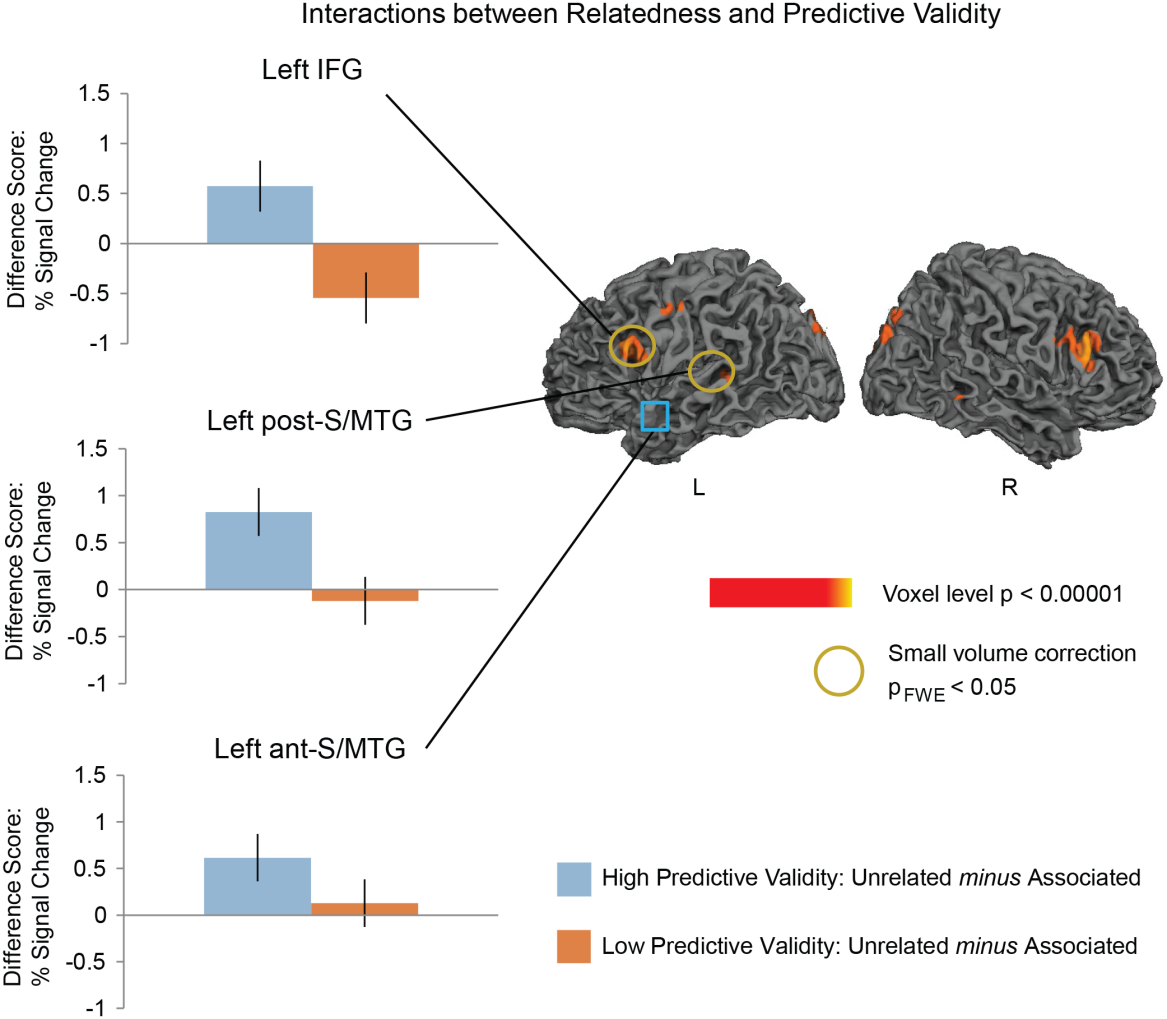
Left: Hemodynamic response suppression effects at each level of Predictive Validity. Graphs show the mean *differences* of the contrast estimates (with standard errors) for Unrelated minus Associated word pairs from the significant peaks in the regions of interest used for small volume correction. Right: Statistical maps showing interactions between Relatedness and Predictive Validity. Effects are shown at a voxel-level significance threshold of p<0.005, k>10. Yellow—red: more activity to Unrelated than Associated word pairs in the higher predictive validity blocks *or* less activity to Unrelated than Associated word pairs in the lower predictive validity blocks. Yellow circles indicate regions that reached a small volume correction FWE-corrected threshold of p<0.05 at the peak of *a priori* regions of interest. The left anterior superior/middle temporal gyrus, indicated with the blue square, was an *a priori* region of interest that did not show a significant interaction between Relatedness and Predictive Validity, although it did show a significant effect of Relatedness in the higher predictive validity blocks.

Follow-up comparisons within the left IFG cluster showed a near-significant priming effect in the higher predictive validity blocks (Z = 2.67, p_FWE_ = 0.054). In the lower predictive validity blocks, there was a trend towards the opposite effect in this region with more activity to the Associated than the Unrelated word pairs—hemodynamic response enhancement (Z = 2.56, p_FWE_ = 0.071), see Fig 3.

Follow-up comparisons in the left post-S/MTG showed a Relatedness priming effect in the higher predictive validity block (Z = 3.37, p_FWE_<0.01), but no effect in the lower predictive validity bock (Z = 1.15, p_FWE_ = 0.58), see Fig 3.

Although in the left ant-S/MTG the interaction between Relatedness and Predictive Validity was not significant, we carried out planned comparisons within this region at each level of Relatedness, given our previous findings using MEG and a preliminary fMRI findings using an FIR model [78,86,87]]. Consistent with our previous findings [51]], we saw an effect of Relatedness in the higher predictive validity block (Z = 3.1, p_FWE_<0.01), but not in the lower predictive validity block (Z<1).

### fMRI functional task-related connectivity analysis

**Fig 4:**
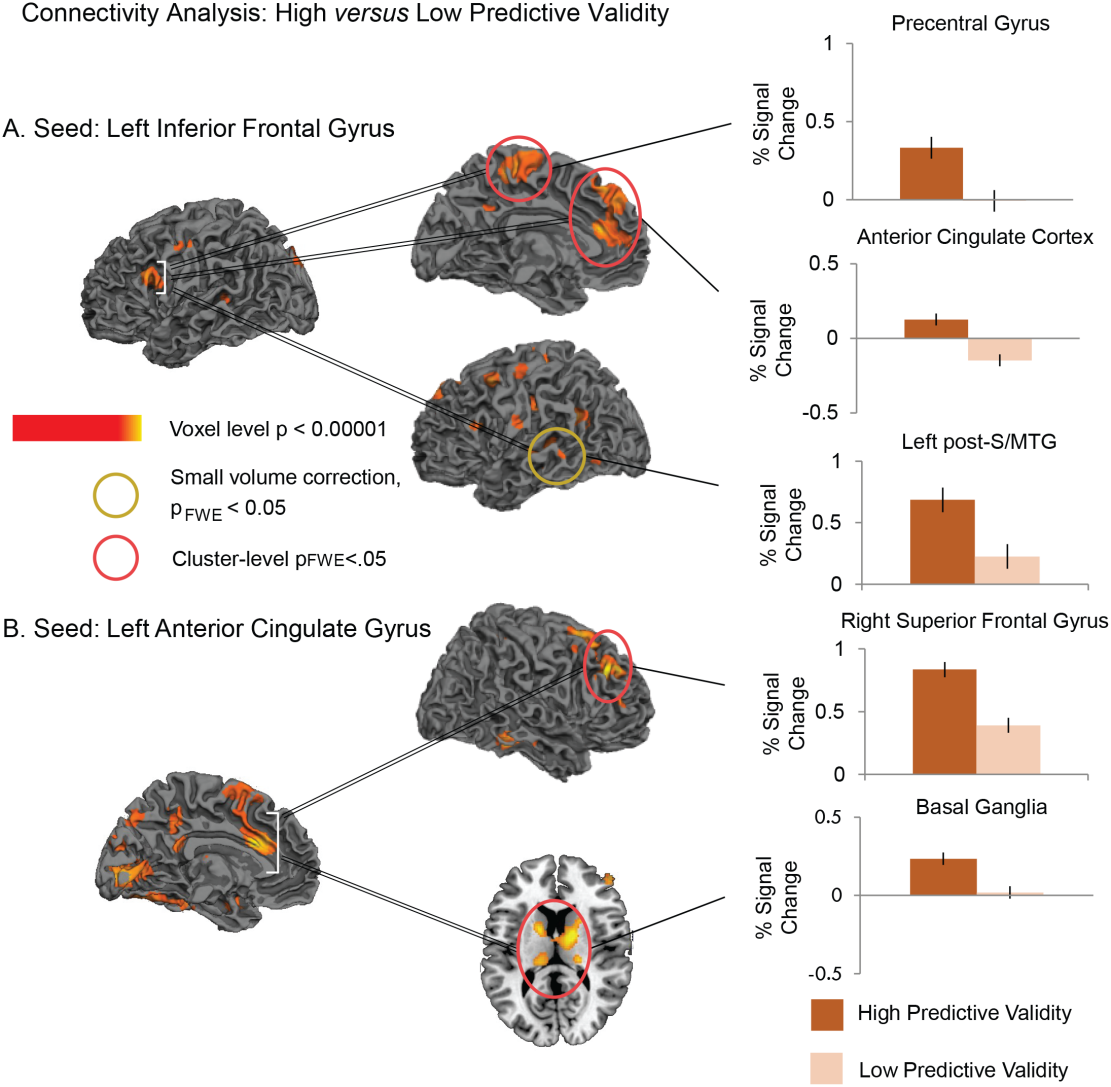
Statistical maps showing increased functional connectivity in the higher predictive validity blocks relative to the lower predictive validity blocks. Seed regions (A: the left inferior frontal gyrus; B: left anterior cingulate gyrus) are indicated with white brackets. Effects are shown at a voxel-level significance threshold of p<0.005, and include clusters consisting of 10 or more contiguous voxels. Yellow—red: More functional connectivity from seed regions in higher predictive validity blocks than Lower predictive validity blocks. Red circles indicate clusters that reached a cluster-level FWE-corrected threshold of p<0.05. Yellow circles indicate regions that reached a small volume correction FWE-corrected threshold of p<0.05 at the peak of *a priori* regions of interest. Graphs show the contrast estimates (and standard errors) from representative peaks within regions that reached cluster or small volume corrected significance. See Table 3 for the full list of peaks.

We first looked at the connectivity patterns from a seed in the left IFG, which was defined based on the functional activation in the left IFG for the interaction between Predictive Validity and Relatedness. We compared connectivity from this seed between the higher and lower predictive validity blocks. This revealed significantly more connectivity under conditions of higher (versus lower) predictive validity to: (a) bilateral anterior cingulate cortex and paracentral gyrus (whole-brain voxel-level, p<0.005, cluster-level FWE-corrected, p<0.05), and (b) left post-S/MTG (significant on small volume correction: Z = 3.03, p_FWE_<0.01), see Fig 4 and Table 3 for full set of coordinates within these clusters. The reverse contrast (lower predictive validity > higher predictive validity) did not reveal any significant effects.

**Table 3:** Increased functional connectivity in the higher predictive validity blocks relative to the Lower predictive validity blocks, from two seed regions: the left inferior frontal gyrus and left anterior cingulate.

Region	R/L	BA	Peak voxel p-value	z-score	MNI (x, y, z)	Cluster extent k	Cluster level p-value (FWE corrected)
**1. Seed Region—Left Inferior Frontal Gyrus**							
**Higher > Lower predictive validity**							
Anterior cingulate cortex	L	24/32	< 0.0001	4.25	-12, 34, 18†	1,452	0.0119
Anterior cingulate cortex	R	24	0.0004	3.37	8, 36, 10		
Superior frontal cortex (medial)	L	9	0.0006	3.22	-4, 46, 40		
Superior frontal cortex (medial)	R	8/9	0.0001	3.64	4, 36, 48		
Precentral gyrus	R	4	0.0003	3.45	14, -24, 66†	1,432	0.0127
Postcentral gyrus	L	3	0.0012	3.04	-28, -36, 58		
**2. Seed Region—Left Anterior Cingulate Cortex**							
**Higher > Lower predictive validity**							
Dorsolateral Prefrontal Cortex (Superior Frontal Gyrus)	L	6	0.0004	3.33	-20, 4, 54	674	0.1657
Dorsolateral Prefrontal Cortex (Middle Frontal Gyrus)		8	0.0007	3.19	-24, 22, 64		
Dorsolateral Prefrontal Cortex (Middle Frontal Gyrus)	R	9	< 0.0001	4.25	32, 36, 34†	1,825	0.003
Dorsolateral Prefrontal Cortex (Superior Frontal Gyrus)		8	0.0001	3.69	26, 18, 60		
Basal ganglia (caudate/putamen)	L	-	0.001	3.08	-26, -10, 20	1,398	0.0119
Basal ganglia (caudate/putamen)	R	-	0.0004	3.39	20, 10, 14†		
Thalamus	L	-	< 0.0001	3.82	-14, 4, 16		
Thalamus	R	-	< 0.0001	3.88	14, -2, 10		

All regions shown reached a cluster-level significance threshold (after family-wise error correction) of *p* < 0.05. Anatomical locations, MNI coordinates, and approximate Brodmann areas (BA) correspond to the p-values and z-scores of representative peaks within each cluster. Both the AAL atlas and the SPM anatomy toolbox [85]] were used to define the anatomical regions reported. Only one peak per anatomical region is reported for each hemisphere. The cluster-level p-values indicate the cluster-level significance after family-wise error correction, and k indicates the number of contiguous voxels within each cluster.

^†^ The contrast estimate for this peak is shown in Fig 4.

We next looked at the connectivity patterns from a seed in the left ACC, which was defined based on the functional activation in this region for the main effect of Relatedness. This revealed significantly more connectivity under conditions of higher (versus lower) predictive validity (whole-brain voxel-level, p<0.005, cluster-level FWE-corrected, p<0.05) to (a) the right superior frontal cortex extending into the middle frontal gyrus as well as the left superior frontal gyrus, and (b) a cluster that extended bilaterally from the thalami into the posterior part of the caudate, palladium and putamen, see Fig 4 and Table 3. Once again, the reverse contrast did not reveal any effects.

## Discussion

In this study we used fMRI with a semantic priming relatedness proportion paradigm to characterize the neuroanatomical regions engaged in semantic prediction and adaptation. This paradigm allowed us to determine how hemodynamic activity was modulated to identical associated and unrelated prime-target pairs under conditions of both higher and lower predictive validity. Across both predictive validity blocks, we observed reduced activity in response to associated compared to unrelated word pairs—hemodynamic response suppression—within bilateral temporal fusiform cortices and the left anterior/middle cingulate and SMA, consistent with some previous fMRI studies of semantic priming [13,88,89,90]]. Under conditions of higher predictive validity we saw significant hemodynamic response suppression in three additional regions—the left ant-S/MTG, the left IFG and the left post-S/MTG. In the latter two regions, the effect differed qualitatively from the effect seen in the lower predictive validity block, driving a significant interaction between relatedness and predictive validity. A functional connectivity analysis showed that, under conditions of higher versus lower predictive validity, the latter two regions were more tightly interconnected with one another, as well as with the ACC. Also under conditions of higher versus lower predictive validity, the left ACC was also more tightly functionally connected with a lateral prefrontal-subcortical network.

### Distinct neurocognitive mechanisms engaged to fulfilled and unfulfilled semantic predictions?

We interpret the hemodynamic response suppression effect within the *left anterior S/MTG* under conditions of higher predictive validity as reflecting facilitated semantic processing of semantically associated targets that *confirmed* prior semantic predictions. This interpretation is based on our recent MEG/ERP study using the same paradigm in an overlapping set of participants [51], which showed that this region was modulated between 350-450ms—the time window that corresponds to the N400, an ERP component that is selectively sensitive to semantic facilitation (e.g. [10,41]). We suggest that, under conditions of higher predictive validity, this region acted as a ‘hub’ that used context in a predictive fashion to facilitate access to semantic representations that were highly distributed across multiple cortical regions [22,23,52,91].

Although the hemodynamic response suppression effect within the left anterior S/MTG was significant in the higher but not in the lower predictive validity blocks, the difference in its modulation across the two blocks (the interaction between relatedness and predictive validity) was not significant. This may be because, as in our previous MEG study [51], this region showed a numerical trend towards a relatedness effect in the lower predictive validity blocks, perhaps reflecting weaker semantic facilitation (see also [24]). On this account, any difference in modulation within this region across the two blocks was quantitative rather than qualitative, and signal loss due to susceptibility artifact in this region may have reduced our power to detect this quantitative difference statistically.

We offer a different interpretation of the hemodynamic response suppression effect observed under conditions of higher predictive validity within the left IFG and posterior portion of the left S/MTG. Neither of these regions showed modulation within the N400 time window in our previous MEG study [51], and we suggest that their modulation was primarily driven by increased activity to the semantically unrelated word-pair trials in which the targets *disconfirmed* prior semantic predictions. More specifically, we suggest that, under conditions of higher predictive validity, the left IFG mediated the top-down *suppression* of semantic features that were predicted on the basis of prime words but that were unfulfilled by unrelated target words, while the left post-S/MTG reflected increased lexico-semantic processing of these unpredicted unrelated targets.

This interpretation of left IFG modulation is based on a large number of fMRI and lesion studies that have implicated this region in suppressing semantic features that act as distractors for performance on a wide variety of tasks, ranging from the disambiguation of word meaning [36–39,54], to cued semantic association [33,92]]. In the present study, we suggest that, by suppressing semantic features that were predicted by primes but unfulfilled by unrelated targets, the increased left IFG activity aided participants’ classification of the unrelated targets’ semantic features, as required by the task. More generally, this interpretation is in line with the proposed role of the ventrolateral prefrontal cortex in aspects of executive function, particularly the selection of a class of contextually relevant information from sets of potential competing distractors to serve a particular goal ([93–96]; see [97,98] for more general reviews of prefrontal function, and see [40] for discussion in relation to language processing). Of particular relevance to the current findings, this account is consistent with previous findings reporting that the left IFG is more active in trials in which words disconfirm highly semantically predictive contexts than to trials with non-predictive contexts [13,35].

In at least some of these previous studies (e.g. during the resolution of ambiguity [39,54] and during semantic priming [13]), the left IFG was co-activated with posterior portions of the left temporal cortex, just as in the present study. We suggest that, in our study, the increased activity within the left post-S/MTG reflected increased demands of *lexico*-semantic processing of target words [21,25–27]. Lexico-semantic processing within the left posterior S/MTG can be dissociated from the more purely semantic function of the left anterior temporal cortex discussed above (see also [99]). More specifically, in the present study, we suggest that the increased activity within the left post-S/MTG reflected the increased demands of mapping word-form (phonological or orthographic) representations of unrelated targets on to their corresponding semantic features, which had not been pre-activated. On this account, the top-down suppression of unfulfilled semantic predictions within the left IFG and bottom up lexico-semantic processing of unrelated targets are functionally linked. This interpretation is in keeping with the assumptions of many connectionist architectures (e.g. [100]) as well as neural frameworks that posit links between these two regions (e.g. [101]). In the present study, it is further supported by our functional connectivity analysis which showed that these two regions were more tightly functionally connected in the higher than the lower predictive validity blocks. This finding is consistent with the well-described structural connections between these two regions through the arcuate fasciculus [102–106] as well as with previous reports that these two regions are tightly interconnected at rest (e.g. [107,108]), and in association with different aspects of language processing, e.g. [109–111]).

Notably, the pattern of modulation within both the left IFG and the left post-S/MTG was somewhat different under conditions of lower predictive validity where there was no hint of hemodynamic response suppression, even at lower thresholds of significance. Indeed, within the left IFG, there was a near-significant reversed priming effect with more activity to associated than unrelated word pairs—so-called *hemodynamic response enhancement* (see [112] for a general review of factors that can contribute to this type of reverse hemodynamic priming effect). We tentatively suggest that this reflected a more reactive strategy of semantically matching the semantic features of prime and target (see [14] for evidence that semantic matching is associated with hemodynamic response enhancement) in order to aid task performance (see also [24] for discussion). On this account, whether we engage or disengage in semantic predictive processing is not only a function of the statistical structure of the wider contextual environment, but also of participants’ specific tasks and goals (see [1], sections 3.4 and 3.5 for discussion): one might therefore expect to see quite different patterns of modulation in association with tasks in which semantic prediction is not necessarily beneficial to performance (see [45,113] for discussion in relation to behavioral findings).

### Adaptation

Our use of the relatedness proportion paradigm also afforded us the opportunity to explore relationships between semantic prediction and adaptation. As noted in the Introduction, prediction and adaptation are reciprocally linked: not only can adaptation to the statistical structure of the broader contextual environment modulate the strength of predictions—the underlying logic of this paradigm—but prediction itself may be the driving force behind adaptation—an idea that is central to theories of classical conditioning [55,56], connectionist learning [57,58] and Bayesian inference and learning [59,60]. The basic idea is that, at any given time, an agent’s graded predictions are compared with new inputs, and any differences between these predictions and the state of the system after these new inputs are encountered—prediction error—are used to update the agent’s knowledge about the statistical contingencies that best explain these inputs (within a connectionist framework, these are encoded as graded connections, and within a Bayesian framework, they can be described as probabilistic beliefs). By iteratively predicting and updating knowledge on the basis of new observations, the agent’s predictions will, over time, become increasingly accurate such that overall prediction error is minimized and the agent’s knowledge accurately reflects the statistical structure of her environment.

Our functional connectivity data provide evidence that, under conditions of higher versus lower predictive validity, regions associated with semantic prediction error (the response to unfulfilled predictions within the left IFG and post-S/MTG) are more tightly connected to a region that is thought to play a critical role in monitoring changes in the statistical contingencies between stimuli or stimulus-response mappings—the ACC (see [63–66]). One possibility is that these tighter functional connections reflected a role of the ACC in using its assessment of the reliability of the agent’s prior knowledge about these mappings to *weight* the degree to which current prediction error (the response to unpredicted target words associated with left IFG and post-S/MTG activity) influenced the rate of adaptation [65,66]. Adaptation (learning) itself may have been mediated by a lateral prefrontal-subcortical network, to which the anterior cingulate was also more functionally interconnected under conditions of higher versus lower predictive validity. This included the superior lateral frontal cortices and subcortical regions (thalamus and basal ganglia), which have previously been implicated in language monitoring [68,69], pattern-based sequential learning [70–72] and adaptation [73].

### Open questions

Our findings raise a number of open questions. One set of questions concerns the relationships between activity within the neuroanatomical regions discussed here and various ERP components that have been associated with confirmed and disconfirmed semantic predictions. As discussed, based on our previous MEG/ERP findings using the same paradigm [51], we interpret the modulation of activity within the anterior temporal cortex in the high predictive validity block as reflecting activity within the N400 time window to *confirmed* semantic predictions. It is tempting to link the modulation of the left IFG (together with the left post-S/MTG) to another ERP component—a more prolonged anteriorly-distributed negativity effect, which, in our ERP study using this same paradigm, was selectively enhanced to targets that *disconfirmed* semantic predictions [41]. Similar to the left IFG, anterior negativities have been linked to the suppression of semantic features that are predicted on the basis of context with medium certainty but that are not fulfilled by target words [9,53,114] (see Methods for explanation of why participants in this study are likely to have predicted upcoming semantic features with medium certainty in the higher predictive validity blocks).

It is important to recognize, however, that several other later (post-N400) ERP components have also been linked to unfulfilled semantic predictions, including a series of late positivity components (see [6,115] for reviews). Late positivities tend to be evoked by inputs that violate high certainty predictions that are generated not only at the level of semantic features, but also at other level(s) of representation (see [5] for discussion). For example, an anteriorly distributed positivity effect is evoked by words that violate or conflict with high certainty predictions about contingencies between semantic features and word-form (strong *lexical* predictions, e.g. [116]), while a posteriorly distributed or P600 effect is evoked by words that violate or conflict with high certainty predictions about contingencies between semantic features and syntactic properties (strong predictions about likely structure [115]). These late positivity effects may be linked to particularly rapid adaptation to new statistical environments. Thus, one possibility, which could be explored in future work, is that they are associated with further recruitment of the anterior cingulate, which, as discussed above, is thought to monitor changes in statistical contingencies in the environment, and indeed was first characterized as monitoring errors or conflicts between pre-potent predictions and bottom-up evidence [66,117,118].

A second set of open questions concerns the relationship between these findings and predictive processing during sentence and discourse processing. As we have discussed, the advantage of the relatedness proportion semantic priming design is that it was able to isolate predictive processing while holding both the local context and target words constant across conditions. However, it is necessarily more artificial than examining prediction during higher-level language comprehension, and here we explored just two levels of predictive validity. It will therefore be important for future studies to determine whether the same set of regions is modulated by predictive constraint in a more graded fashion during sentence and discourse-level processing.

### Conclusions

We have shown clear differences in the modulation of activity within left temporal and inferior frontal cortices to the same associated and unrelated context prime-target pairs under conditions of higher versus lower predictive validity. Based on these results, we have suggested that the anterior superior/middle temporal cortex plays a role in predictive semantic facilitation, while the posterior superior/middle temporal cortex and the left inferior frontal cortex together mediate the suppression of unfulfilled medium-certainty semantic predictions and the lexico-semantic processing of unpredicted inputs, respectively. We have also shown that, under conditions of higher predictive validity, the latter two regions were not only more tightly interconnected with one another, but also with the anterior cingulate cortex, which, in turn was more tightly connected with a lateral prefrontal-subcortical network. This is consistent with a role of the anterior cingulate in mediating between prediction error and adaptation. This work therefore paves the way towards understanding how our brains use prediction error to adapt to our ever-changing real-world communicative environments.

## References

[1] Kuperberg GR, Jaeger TF (2015) What do we mean by prediction in language comprehension? Language, Cognition, and Neuroscience 31: 32–59. 10.1080/23273798.2015.1102299 PMC4850025 27135040

[2] Jaeger TF, Snider NE (2013) Alignment as a consequence of expectation adaptation: syntactic priming is affected by the prime’s prediction error given both prior and recent experience. Cognition 127: 57–83. 10.1016/j.cognition.2012.10.013 23354056 PMC7313543

[3] Fine AB, Jaeger TF, Farmer TA, Qian T (2013) Rapid expectation adaptation during syntactic comprehension. PLoS One 8: e77661 10.1371/journal.pone.0077661 24204909 PMC3813674

[4] Kleinschmidt DF, Jaeger FT (2015) Robust speech perception: Recognize the familiar, generalize to the similar, and adapt to the novel. Psychological Review 122: 148–203. 10.1037/a0038695 25844873 PMC4744792

[5] Kuperberg GR (2013) The proactive comprehender: What event-related potentials tell us about the dynamics of reading comprehension In: Miller B, Cutting L, McCardle P, editors. Unraveling Reading Comprehension: Behavioral, Neurobiological, and Genetic Components. Baltimore, MD: Paul Brookes Publishing pp. 176–192.

[6] Van Petten C, Luka BJ (2012) Prediction during language comprehension: benefits, costs, and ERP components. International Journal of Psychophysiology 83: 176–190. 10.1016/j.ijpsycho.2011.09.015 22019481

[7] Kutas M, DeLong KA, Smith NJ (2011) A look around at what lies ahead: Prediction and predictability in language processing In: Bar M, editor. Predictions in the brain: Using our past to generate a future. New York: Oxford University Press pp. 190–207.

[8] DeLong KA, Urbach TP, Kutas M (2005) Probabilistic word pre-activation during language comprehension inferred from electrical brain activity. Nature Neuroscience 8: 1117–1121. 16007080 10.1038/nn1504

[9] Wlotko EW, Federmeier KD (2012) So that's what you meant! Event-related potentials reveal multiple aspects of context use during construction of message-level meaning. NeuroImage 62: 356–366. 10.1016/j.neuroimage.2012.04.054 22565202 PMC3457057

[10] Federmeier KD (2007) Thinking ahead: the role and roots of prediction in language comprehension. Psychophysiology 44: 491–505. 17521377 10.1111/j.1469-8986.2007.00531.x PMC2712632

[11] DeLong KA, Troyer M, Kutas M (2014) Pre-processing in sentence comprehension: sensitivity to likely upcoming meaning and structure. Language and Linguistics Compass 8: 631–645. 27525035 10.1111/lnc3.12093 PMC4982702

[12] Kotz SA, Cappa SF, von Cramon DY, Friederici AD (2002) Modulation of the lexical-semantic network by auditory semantic priming: An event-related functional MRI study. NeuroImage 17: 1761–1772. 12498750 10.1006/nimg.2002.1316

[13] Gold BT, Balota DA, Jones SJ, Powell DK, Smith CD, Andersen AH (2006) Dissociation of automatic and strategic lexical-semantics: Functional magnetic resonance imaging evidence for differing roles of multiple frontotemporal regions. Journal of Neuroscience 26: 6523–6532. 16775140 10.1523/JNEUROSCI.0808-06.2006 PMC6674026

[14] Kuperberg GR, Lakshmanan BM, Greve DN, West WC (2008) Task and semantic relationship influence both the polarity and localization of hemodynamic modulation during lexico-semantic processing. Human Brain Mapping 29: 544–561. 17674356 10.1002/hbm.20419 PMC3141820

[15] Baumgaertner A, Weiller C, Buchel C (2002) Event-related fMRI reveals cortical sites involved in contextual sentence integration. NeuroImage 16: 736–745. 12169257 10.1006/nimg.2002.1134

[16] Kuperberg GR, Sitnikova T, Caplan D, Holcomb PJ (2003) Electrophysiological distinctions in processing conceptual relationships within simple sentences. Cognitive Brain Research 17: 117–129. 12763198 10.1016/s0926-6410(03)00086-7

[17] Hagoort P, Hald L, Bastiaansen M, Petersson KM (2004) Integration of word meaning and world knowledge in language comprehension. Science 304: 438–441. 15031438 10.1126/science.1095455

[18] Kuperberg GR, Sitnikova T, Lakshmanan BM (2008) Neuroanatomical distinctions within the semantic system during sentence comprehension: evidence from Functional Magnetic Resonance Imaging. NeuroImage 40: 367–388. 10.1016/j.neuroimage.2007.10.009 18248739 PMC3141816

[19] Dien J, Franklin MS, Michelson CA, Lemen LC, Adams CL, Kiehl KA (2008) fMRI characterization of the language formulation area. Brain Research 1229: 179–192. 10.1016/j.brainres.2008.06.107 18639536

[20] Kuperberg GR, Lakshmanan BM, Caplan DN, Holcomb PJ (2006) Making sense of discourse: an fMRI study of causal inferencing across sentences. NeuroImage 33: 343–361. 16876436 10.1016/j.neuroimage.2006.06.001

[21] Lau EF, Phillips C, Poeppel D (2008) A cortical network for semantics: (De)constructing the N400. Nature Reviews Neuroscience 9: 920–933. 10.1038/nrn2532 19020511

[22] Patterson K, Nestor PJ, Rogers TT (2007) Where do you know what you know? The representation of semantic knowledge in the human brain. Nature Reviews Neuroscience 8: 976–987. 18026167 10.1038/nrn2277

[23] Price CJ (2012) A review and synthesis of the first 20 years of PET and fMRI studies of heard speech, spoken language and reading. NeuroImage 62: 816–847. 10.1016/j.neuroimage.2012.04.062 22584224 PMC3398395

[24] Lau EF, Gramfort A, Hämäläinen MS, Kuperberg GR (2013) Automatic semantic facilitation in anterior temporal cortex revealed through multimodal neuroimaging. Journal of Neuroscience 33: 17174–17181. 10.1523/JNEUROSCI.1018-13.2013 24155321 PMC3807034

[25] Hickok G, Poeppel D (2007) The cortical organization of speech processing. Nature Reviews Neuroscience 8: 393–402. 17431404 10.1038/nrn2113

[26] Martin A (2007) The representation of object concepts in the brain. Annual Review of Psychology 58: 25–45. 16968210 10.1146/annurev.psych.57.102904.190143

[27] Binder JR, Desai RH, Graves WW, Conant LL (2009) Where is the semantic system? A critical review and meta-analysis of 120 functional neuroimaging studies. Cerebral Cortex 19: 2767–2796. 10.1093/cercor/bhp055 19329570 PMC2774390

[28] Milberg W, Blumstein SE, Dworetzky B (1987) Processing of lexical ambiguities in aphasia. Brain and Language 31: 138–150. 2437994 10.1016/0093-934x(87)90065-4

[29] Swaab TY, Brown C, Hagoort P (1998) Understanding ambiguous words in sentence contexts: electrophysiological evidence for delayed contextual selection in Broca's aphasia. Neuropsychologia 36: 737–761. 9751439 10.1016/s0028-3932(97)00174-7

[30] Bedny M, Hulbert JC, Thompson-Schill SL (2007) Understanding words in context: the role of Broca's area in word comprehension. Brain Research 1146: 101–114. 17123486 10.1016/j.brainres.2006.10.012

[31] Robinson G, Blair J, Cipolotti L (1998) Dynamic aphasia: an inability to select between competing verbal responses? Brain 121: 77–89. 9549489 10.1093/brain/121.1.77

[32] Robinson G, Shallice T, Cipolotti L (2005) A failure of high level verbal response selection in progressive dynamic aphasia. Cognitive Neuropsychology 22: 661–694. 10.1080/02643290442000239 21038272

[33] Thompson-Schill SL, D'Esposito M, Aguirre GK, Farah MJ (1997) Role of left inferior prefrontal cortex in retrieval of semantic knowledge: a reevaluation. Proceedings of the National Academy of Sciences 94: 14792–14797. 10.1073/pnas.94.26.14792 PMC25116 9405692

[34] Thompson-Schill SL, D'Esposito M, Kan IP (1999) Effects of repetition and competition on activity in left prefrontal cortex during word generation. Neuron 23: 513–522. 10433263 10.1016/s0896-6273(00)80804-1

[35] Cardillo ER, Aydelott J, Matthews PM, Devlin JT (2004) Left inferior prefrontal cortex activity reflects inhibitory rather than facilitatory priming. Journal of Cognitive Neuroscience 16: 1552–1561. 15601518 10.1162/0898929042568523 PMC2651466

[36] Bedny M, McGill M, Thompson-Schill SL (2008) Semantic adaptation and competition during word comprehension. Cerebral Cortex 18: 2574–2585. 10.1093/cercor/bhn018 18308708 PMC2567420

[37] Mason RA, Just MA (2007) Lexical ambiguity in sentence comprehension. Brain Research 1146: 115–127. 17433891 10.1016/j.brainres.2007.02.076 PMC2713009

[38] Zempleni MZ, Renken R, Hoeks JC, Hoogduin JM, Stowe LA (2007) Semantic ambiguity processing in sentence context: Evidence from event-related fMRI. NeuroImage 34: 1270–1279. 17142061 10.1016/j.neuroimage.2006.09.048

[39] Rodd JM, Johnsrude IS, Davis MH (2012) Dissociating frontotemporal contributions to semantic ambiguity resolution in spoken sentences. Cerebral Cortex 22: 1761–1773. 10.1093/cercor/bhr252 21968566

[40] Novick JM, Trueswell JC, Thompson-Schill SL (2010) Broca’s area and language processing: Evidence for the cognitive control connection. Language and Linguistics Compass 4: 906–924.

[41] Lau EF, Holcomb PJ, Kuperberg GR (2013) Dissociating N400 effects of prediction from association in single-word contexts. Journal of Cognitive Neuroscience 25: 484–502. 10.1162/jocn_a_00328 23163410 PMC3657387

[42] de Groot AMB (1984) Primed lexical decision: Combined effects of the proportion of related prime-target pairs and the stimulus-onset asynchrony of prime and target. Quarterly Journal of Experimental Psychology A: Human Experimental Psychology 36: 253–280.

[43] den Heyer K (1985) On the nature of the proportion effect in semantic priming. Acta Psychologica 60: 25–38.

[44] Seidenberg MS, Waters GS, Sanders M, Langer P (1984) Pre- and postlexical loci of contextual effects on word recognition. Memory & Cognition 12: 315–328. 6503694 10.3758/bf03198291

[45] Keefe DE, Neely JH (1990) Semantic priming in the pronunciation task: The role of prospective prime-generated expectancies. Memory & Cognition 18: 289–298. 2355858 10.3758/bf03213882

[46] Tweedy JR, Lapinski RH, Schvaneveldt RW (1977) Semantic-context effects on word recognition: Influence of varying the proportion of items presented in an appropriate context. Memory & Cognition 5: 84–89. 21331872 10.3758/BF03209197

[47] Neely JH (1991) Semantic priming effects in visual word recognition: A selective review of current findings and theories In: Besner D, Humphreys G.W., editor. Basic Processes in Reading and Visual Word Recognition. Hillsdale, NJ: Erlbaum pp. 264–333.

[48] Holcomb PJ (1988) Automatic and attentional processing: an event-related brain potential analysis of semantic priming. Brain and Language 35: 66–85. 3179703 10.1016/0093-934x(88)90101-0

[49] Brown CM, Hagoort P, Chwilla DJ (2000) An event-related brain potential analysis of visual word priming effects. Brain and Language 72: 158–190. 10722786 10.1006/brln.1999.2284

[50] Norris D, McQueen JM (2008) Shortlist B: a Bayesian model of continuous speech recognition. Psychological Review 115: 357–395. 10.1037/0033-295X.115.2.357 18426294

[51] Lau EF, Weber K, Gramfort A, Hamalainen MS, Kuperberg GR (2014) Spatiotemporal signatures of lexico-semantic prediction. Cerebral Cortex. 10.1093/cercor/bhu219 PMC4785937 25316341

[52] Mummery CJ, Patterson K, Price CJ, Ashburner J, Frackowiak RS, Hodges JR (2000) A voxel-based morphometry study of semantic dementia: Relationship between temporal lobe atrophy and semantic memory. Annals of Neurology 47: 36–45. 10632099

[53] Wlotko EW, Federmeier KD (2012) Age-related changes in the impact of contextual strength on multiple aspects of sentence comprehension. Psychophysiology 49: 770–785. 10.1111/j.1469-8986.2012.01366.x 22469362 PMC4001119

[54] Rodd JM, Davis MH, Johnsrude IS (2005) The neural mechanisms of speech comprehension: fMRI studies of semantic ambiguity. Cerebral Cortex 15: 1261–1269. 15635062 10.1093/cercor/bhi009

[55] Rescorla RA, Wagner AR (1972) A theory of Pavlovian conditioning: Variations in the effectiveness of reinforcement and nonreinforcement In: Prokasy WE, Black AH, editors. Classical conditioning II: Current research and theory. New York: Appleton- Century-Crofts pp. 64–99.

[56] Kruschke JK (2008) Bayesian approaches to associative learning: From passive to active learning. Learning and Behavior 36: 210–226. 18683466 10.3758/lb.36.3.210

[57] Rumelhart DE, Hinton GE, Williams RJ (1986) Learning internal representations by error propagation In: Rumelhart DE, McClelland JL, Group PR, editors. Parallel Distributed Processing: Explorations in the Microstructure of Cognition Vol 1: Foundations. Cambridge, MA: MIT Press pp. 318–362.

[58] Elman JL (1990) Finding structure in time. Cognitive Science 14: 179–211.

[59] Griffiths TL, Kemp C, Tenenbaum JB (2008) Bayesian models of cognition. In: Sun R, editor. The Cambridge Handbook of Computational Psychology. New York: Cambridge University Press pp. 59–100.

[60] Perfors A, Tenenbaum JB, Griffiths TL, Xu F (2011) A tutorial introduction to Bayesian models of cognitive development. Cognition 120: 302–321. 10.1016/j.cognition.2010.11.015 21269608

[61] Pearce JM, Hall G (1980) A model for Pavlovian learning: Variations in the effectiveness of conditioned but not of unconditioned stimuli. Psychological Review 87: 532–552. 7443916

[62] Courville AC, Daw ND, Touretzky DS (2006) Bayesian theories of conditioning in a changing world. Trends in Cognitive Sciences 10: 294–300. 16793323 10.1016/j.tics.2006.05.004

[63] Botvinick MM, Cohen JD, Carter CS (2004) Conflict monitoring and anterior cingulate cortex: an update. Trends in Cognitive Sciences 8: 539–546. 15556023 10.1016/j.tics.2004.10.003

[64] Rushworth MF, Walton ME, Kennerley SW, Bannerman DM (2004) Action sets and decisions in the medial frontal cortex. Trends in Cognitive Sciences 8: 410–417. 15350242 10.1016/j.tics.2004.07.009

[65] Behrens TEJ, Woolrich MW, Walton ME, Rushworth MFS (2007) Learning the value of information in an uncertain world. Nature Neuroscience 10: 1214–1221. 17676057 10.1038/nn1954

[66] Ide JS, Shenoy P, Yu AJ, Li CS (2013) Bayesian prediction and evaluation in the anterior cingulate cortex. Journal of Neuroscience 33: 2039–2047. 10.1523/JNEUROSCI.2201-12.2013 23365241 PMC3711643

[67] Kerns JG, Cohen JD, MacDonald AW 3rd, Cho RY, Stenger VA, Carter CS (2004) Anterior cingulate conflict monitoring and adjustments in control. Science 303: 1023–1026. 14963333 10.1126/science.1089910

[68] Munte TF, Kutas M (2008) Capitalizing on deep brain stimulation: thalamus as a language monitor. Neuron 59: 677–679. 10.1016/j.neuron.2008.08.015 18786350 PMC2642975

[69] Wahl M, Marzinzik F, Friederici AD, Hahne A, Kupsch A, Schneider GH, et al (2008) The human thalamus processes syntactic and semantic language violations. Neuron 59: 695–707. 10.1016/j.neuron.2008.07.011 18786354

[70] Kotz SA, Schwartze M, Schmidt-Kassow M (2009) Non-motor basal ganglia functions: a review and proposal for a model of sensory predictability in auditory language perception. Cortex 45: 982–990. 10.1016/j.cortex.2009.02.010 19361785

[71] Osterhout L, Kim A, Kuperberg GR (2012) The neurobiology of sentence comprehension In: Spivey M, Joannisse M, McRae K, editors. The Cambridge Handbook of Psycholinguistics. Cambridge: Cambridge University Press pp. 365–389.

[72] Dominey PF, Inui T (2009) Cortico-striatal function in sentence comprehension: insights from neurophysiology and modeling. Cortex 45: 1012–1018. 10.1016/j.cortex.2009.03.007 19446801

[73] Erb J, Henry MJ, Eisner F, Obleser J (2013) The brain dynamics of rapid perceptual adaptation to adverse listening conditions. Journal of Neuroscience 33: 10688–10697. 10.1523/JNEUROSCI.4596-12.2013 23804092 PMC6618499

[74] Friston KJ, Buechel C, Fink GR, Morris J, Rolls E, Dolan RJ (1997) Psychophysiological and modulatory interactions in neuroimaging. NeuroImage 6: 218–229. 9344826 10.1006/nimg.1997.0291

[75] Gitelman DR, Penny WD, Ashburner J, Friston KJ (2003) Modeling regional and psychophysiologic interactions in fMRI: the importance of hemodynamic deconvolution. NeuroImage 19: 200–207. 12781739 10.1016/s1053-8119(03)00058-2

[76] Oldfield RC (1971) The assessment and analysis of handedness: the Edinburgh inventory. Neuropsychologia 9: 97–113. 5146491 10.1016/0028-3932(71)90067-4

[77] Nelson DL, McEvoy CL, Schreiber TA (2004) The University of South Florida free association, rhyme, and word fragment norms. Behavior Research Methods, Instruments, & Computers 36: 402–407. 10.3758/bf03195588 15641430

[78] Burock MA, Dale AM (2000) Estimation and detection of event-related fMRI signals with temporally correlated noise: A statistically efficient and unbiased approach. Human Brain Mapping 11: 249–260. 11144754 10.1002/1097-0193(200012)11:4<249::AID-HBM20>3.0.CO;2-5 PMC6872094

[79] Mazaika PK, Hoeft F, Glover GH, Reiss AL (2009) ArtRepair. Center for Interdisciplinary Brain Sciences Research, Stanford University.

[80] Mozes S, Whitfield-Gabrieli S (2011) Artifact Detection Toolbox (ART). MIT: Gabrieli Laboratory.

[81] Henson RN, Rugg MD (2001) Effects of stimulus repetition on latency of the BOLD impulse response. NeuroImage 13: 683.

[82] Worsley KJ, Marrett S, Neelin P, Vandal AC, Friston KJ, Evans AC (1996) A unified statistical approach for determining significant signals in images of cerebral activation. Human Brain Mapping 4: 58–73. 20408186 10.1002/(SICI)1097-0193(1996)4:1<58::AID-HBM4>3.0.CO;2-O

[83] Yarkoni T, Poldrack RA, Nichols TE, Van Essen DC, Wager TD (2011) Large-scale automated synthesis of human functional neuroimaging data. Nature Methods 8: 665–670. 10.1038/nmeth.1635 21706013 PMC3146590

[84] McLaren DG, Ries ML, Xu G, Johnson SC (2012) A generalized form of context-dependent psychophysiological interactions (gPPI): a comparison to standard approaches. NeuroImage 61: 1277–1286. 10.1016/j.neuroimage.2012.03.068 22484411 PMC3376181

[85] Eickhoff SB, Stephan KE, Mohlberg H, Grefkes C, Fink GR, Amunts K, et al (2005) A new SPM toolbox for combining probabilistic cytoarchitectonic maps and functional imaging data. NeuroImage 25: 1325–1335. 15850749 10.1016/j.neuroimage.2004.12.034

[86] Burock MA, Buckner RL, Woldorff MG, Rosen BR, Dale AM (1998) Randomized event-related experimental designs allow for extremely rapid presentation rates using functional MRI. NeuroReport 9: 3735–3739. 9858388 10.1097/00001756-199811160-00030

[87] Dale AM (1999) Optimal experimental design for event-related fMRI. Human Brain Mapping 8: 109–114. 10524601 10.1002/(SICI)1097-0193(1999)8:2/3<109::AID-HBM7>3.0.CO;2-W PMC6873302

[88] Wheatley T, Weisberg J, Beauchamp MS, Martin A (2005) Automatic priming of semantically related words reduces activity in the fusiform gyrus. Journal of Cognitive Neuroscience 17: 1871–1885. 16356325 10.1162/089892905775008689

[89] Rossell SL, Bullmore ET, Williams SC, David AS (2001) Brain activation during automatic and controlled processing of semantic relations: A priming experiment using lexical-decision. Neuropsychologia 39: 1167–1176. 11527554 10.1016/s0028-3932(01)00049-5

[90] Ulrich M, Hoenig K, Gron G, Kiefer M (2013) Brain activation during masked and unmasked semantic priming: commonalities and differences. Journal of Cognitive Neuroscience 25: 2216–2229. 10.1162/jocn_a_00449 23859642

[91] Visser M, Jefferies E, Lambon Ralph MA (2010) Semantic processing in the anterior temporal lobes: a meta-analysis of the functional neuroimaging literature. Journal of Cognitive Neuroscience 22: 1083–1094. 10.1162/jocn.2009.21309 19583477

[92] Badre D, Poldrack RA, Pare-Blagoev EJ, Insler RZ, Wagner AD (2005) Dissociable controlled retrieval and generalized selection mechanisms in ventrolateral prefrontal cortex. Neuron 47: 907–918. 16157284 10.1016/j.neuron.2005.07.023

[93] Badre D (2008) Cognitive control, hierarchy, and the rostro-caudal organization of the frontal lobes. Trends in Cognitive Sciences 12: 193–200. 10.1016/j.tics.2008.02.004 18403252

[94] Badre D, D'Esposito M (2009) Is the rostro-caudal axis of the frontal lobe hierarchical? Nature Reviews Neuroscience 10: 659–669. 10.1038/nrn2667 19672274 PMC3258028

[95] Hon N, Ong J, Tan R, Yang TH (2012) Different types of target probability have different prefrontal consequences. NeuroImage 59: 655–662. 10.1016/j.neuroimage.2011.06.093 21803165

[96] Petrides M (2005) Lateral prefrontal cortex: architectonic and functional organization. Philosophical Transactions of the Royal Society of London Series B: Biological Sciences 360: 781–795. 15937012 10.1098/rstb.2005.1631 PMC1569489

[97] Miller EK, Cohen JD (2001) An integrative theory of prefrontal cortex function. Annual Review of Neuroscience 24: 167–202. 11283309 10.1146/annurev.neuro.24.1.167

[98] Petrides M (2000) Mapping prefrontal cortical systems for the control of cognition In: Toga AW, Mazziotta JC, editors. Brain Mapping: The Systems: Academic Press pp. 159–176.

[99] Rissman J, Eliassen JC, Blumstein SE (2003) An event-related fMRI investigation of implicit semantic priming. Journal of Cognitive Neuroscience 15: 1160–1175. 14709234 10.1162/089892903322598120

[100] Rogers TT, McClelland JL (2008) Précis of semantic cognition: A parallel distributed processing approach. Behavioral and Brain Sciences 31: 689–749.

[101] Baggio G, Hagoort P (2011) The balance between memory and unification in semantics: A dynamic account of the N400. Language and Cognitive Processes 26: 1338–1367.

[102] Catani M, Allin MP, Husain M, Pugliese L, Mesulam MM, Murray RM, et al (2007) Symmetries in human brain language pathways correlate with verbal recall. Proceedings of the National Academy of Sciences 104: 17163–17168. 10.1073/pnas.0702116104 PMC2040413 17939998

[103] Catani M, Jones DK, Ffytche DH (2005) Perisylvian language networks of the human brain. Annals of Neurology 57: 8–16. 15597383 10.1002/ana.20319

[104] Glasser MF, Rilling JK (2008) DTI tractography of the human brain's language pathways. Cerebral Cortex 18: 2471–2482. 10.1093/cercor/bhn011 18281301

[105] Rilling JK, Glasser MF, Preuss TM, Ma X, Zhao T, Hu X, et al (2008) The evolution of the arcuate fasciculus revealed with comparative DTI. Nat Neurosci 11: 426–428. 10.1038/nn2072 18344993

[106] Déjèrine JJ (1901) Anatomie des centres nerveux. Paris: Rueff et Cie.

[107] Xiang HD, Fonteijn HM, Norris DG, Hagoort P (2010) Topographical functional connectivity pattern in the perisylvian language networks. Cerebral Cortex 20: 549–560. 10.1093/cercor/bhp119 19546155

[108] Turken AU, Dronkers NF (2011) The neural architecture of the language comprehension network: converging evidence from lesion and connectivity analyses. Frontiers in systems neuroscience 5: 1 10.3389/fnsys.2011.00001 21347218 PMC3039157

[109] Snijders TM, Petersson KM, Hagoort P (2010) Effective connectivity of cortical and subcortical regions during unification of sentence structure. NeuroImage 52: 1633–1644. 10.1016/j.neuroimage.2010.05.035 20493954

[110] Papoutsi M, Stamatakis EA, Griffiths J, Marslen-Wilson WD, Tyler LK (2011) Is left fronto-temporal connectivity essential for syntax? Effective connectivity, tractography and performance in left-hemisphere damaged patients. NeuroImage 58: 656–664. 10.1016/j.neuroimage.2011.06.036 21722742

[111] den Ouden DB, Saur D, Mader W, Schelter B, Lukic S, Wali E, et al (2012) Network modulation during complex syntactic processing. NeuroImage 59: 815–823. 10.1016/j.neuroimage.2011.07.057 21820518 PMC3195988

[112] Segaert K, Weber K, de Lange FP, Petersson KM, Hagoort P (2013) The suppression of repetition enhancement: A review of fMRI studies. Neuropsychologia 51: 59–66. 10.1016/j.neuropsychologia.2012.11.006 23159344

[113] Neely JH, Keefe DE, Ross K (1989) Semantic priming in the lexical decision task: Roles of prospective prime-generated expectancies and retrospective semantic matching. Journal of Experimental Psychology: Learning, Memory, and Cognition 15: 1003–1019. 2530303 10.1037//0278-7393.15.6.1003

[114] Lee CL, Federmeier KD (2006) To mind the mind: an event-related potential study of word class and semantic ambiguity. Brain Research 1081: 191–202. 16516169 10.1016/j.brainres.2006.01.058 PMC2728580

[115] Kuperberg GR (2007) Neural mechanisms of language comprehension: Challenges to syntax. Brain Research 1146: 23–49. 17400197 10.1016/j.brainres.2006.12.063

[116] Federmeier KD, Wlotko EW, De Ochoa-Dewald E, Kutas M (2007) Multiple effects of sentential constraint on word processing. Brain Research 1146: 75–84. 16901469 10.1016/j.brainres.2006.06.101 PMC2704150

[117] Botvinick MM, Braver TS, Barch DM, Carter CS, Cohen JD (2001) Conflict monitoring and cognitive control. Psychological Review 108: 624–652. 11488380 10.1037/0033-295x.108.3.624

[118] Yu AJ, Dayan P, Cohen JD (2009) Dynamics of attentional selection under conflict: toward a rational Bayesian account. Journal of Experimental Psychology: Human Perception and Performance 35: 700–717. 10.1037/a0013553 19485686 PMC3432507

